# A Review on Molecularly Imprinted Polymers Preparation by Computational Simulation-Aided Methods

**DOI:** 10.3390/polym13162657

**Published:** 2021-08-10

**Authors:** Zhimin Liu, Zhigang Xu, Dan Wang, Yuming Yang, Yunli Duan, Liping Ma, Tao Lin, Hongcheng Liu

**Affiliations:** 1 Faculty of Environmental Science and Engineering, Kunming University of Science and Technology, Kunming 650500, China; lab_chem@126.com; 2 Faculty of Science, Kunming University of Science and Technology, Kunming 650500, China; yangym1205@163.com (Y.Y.); chemdyl@163.com (Y.D.); 3 Institute of Quality Standard and Testing Technology, Yunnan Academy of Agriculture Science, Kunming 650223, China; lintaonj@126.com (T.L.); liuorg@163.com (H.L.)

**Keywords:** computational simulation, molecularly imprinted polymers, intermolecular interaction

## Abstract

Molecularly imprinted polymers (MIPs) are obtained by initiating the polymerization of functional monomers surrounding a template molecule in the presence of crosslinkers and porogens. The best adsorption performance can be achieved by optimizing the polymerization conditions, but this process is time consuming and labor-intensive. Theoretical calculation based on calculation simulations and intermolecular forces is an effective method to solve this problem because it is convenient, versatile, environmentally friendly, and inexpensive. In this article, computational simulation modeling methods are introduced, and the theoretical optimization methods of various molecular simulation calculation software for preparing molecularly imprinted polymers are proposed. The progress in research on and application of molecularly imprinted polymers prepared by computational simulations and computational software in the past two decades are reviewed. Computer molecular simulation methods, including molecular mechanics, molecular dynamics and quantum mechanics, are universally applicable for the MIP-based materials. Furthermore, the new role of computational simulation in the future development of molecular imprinting technology is explored.

## 1. Introduction

**Figure 1:**
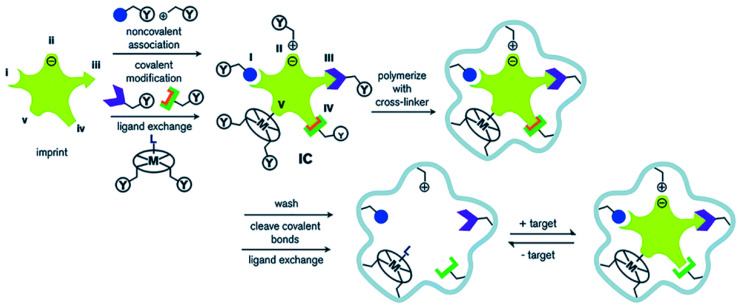
Schematic diagram of the molecular imprinting process: (**I**) non-covalent, (**II**) electrostatic/ionic, (**III**) covalent, (**IV**) semi-covalent, and (**V**) coordination to a metal center (Reprinted with permission from [22]. Copyright 2014 Royal Society of Chemistry).

**Figure 2:**
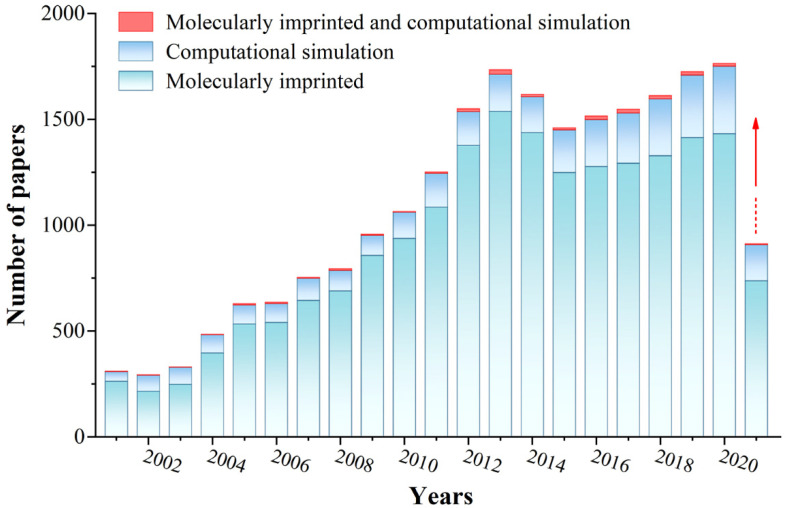
The literature statistics of MIPs and computational simulation. (Database: Scifinder; Search keywords: molecularly imprinted, computational simulation, molecularly imprinted and computational simulation, respectively. Search time: 13 June 2021).

Molecularly imprinted polymers (MIPs) are porous materials with specific recognition capacity towards the template molecule, which are obtained by self-assembly of template molecules and functional monomers in a porogen, and then polymerization is initiated in the presence of a cross-linking agent. The process of preparing MIPs is outlined in Figure 1. When the template molecule interacts with the functional monomer, the imprinting site is memorized through multiple action effects and fixed through the polymerization process. After the template is removed, the adsorption cavity complementary in shape and structure to the template molecule is left in the polymer matrix, which can selectively recognize the target molecule. Molecular imprinting technology originated from antibody immunology, that is, the specific combination of “lock and key” between antibody and antigen [1]. In 1973, Wulff [2] prepared organic MIPs for the first time. Since then, MIPs have attracted widespread attention. At present, MIPs, as a kind of intelligent adsorption material, are widely used in various fields, such as chromatographic separation [3], solid phase extraction [4,5,6], sensors [7,8,9], and biomedicine [10,11]. In the past two decades, great progress in MIPs has been achieved (Figure 2). A variety of novel and interesting imprinted polymers, including supramolecular imprinted polymers [12,13], multitemplate imprinted polymers [14,15], multifunctional monomer imprinted polymers [16,17], dummy template imprinted polymers [18,19], and chiral recognition polymers [20,21], have been developed. In fact, synthesis parameters have been obtained through experimental optimization in most cases. Finding complex and cumbersome conditions is time consuming and laborious. Moreover, numerous organic reagents are used. These factors severely restrict the application and promotion of molecular imprinting technology.

Computational simulation has rapidly developed in recent years. It uses computer technology as a carrier and combines the theoretical basis of quantum mechanics and statistical mechanics as a tool-based cross-discipline. Molecular simulation calculation employs computer technology to simulate changes in the static structure and dynamic motion of molecules by calculating and comparing the relationship between the form and energy of the interaction between molecules to effectively explain the mechanism of action at the molecular level. The method is simple to operate and not restricted by the space environment, and the calculation is accurate and efficient. At present, many reports on the application of computational simulation in molecular imprinting technology have been published [23,24,25,26]. Computational simulation greatly reduces the cost of condition optimization during the polymerization of MIPs. Furthermore, it can effectively predict the more stable conformational composition between the template and the monomer. It can even simulate and calculate the types of porogens, crosslinkers, and initiators [27,28]. In this paper, the theoretical methods for simulating MIPs are briefly summarized, and the progress in the application of MIP simulation in molecular imprinting technology over the past 20 years has been reviewed. This provides insights into the cross application of molecular imprinting technology and computational simulation and the development of green chemistry.

## 2. Theoretical Methods of Computational Simulation for MIPs

The methods used in the theoretical calculation and simulation of various MIP designs are molecular mechanics (MM), molecular dynamics (MD), and quantum mechanics (QM). The computational cost of MM optimization is considerably lower than that of QM, and thus it is orders of magnitude faster than the latter. However, the accuracy of MM results is limited by simplified calculation models, which allow the reduction in calculation costs. The QM approach can better solve the problem of choosing the appropriate initial direction of interacting molecules because it is more accurate than the other methods. However, the computational complexity of the QM approach exponentially increases as the number of molecules involved in the calculation system increases. The MD method can effectively address this problem. When simulating the dynamic process of the interaction between molecules, changes in the molecule itself are often not considered, thereby making the calculation of the simulation method more efficient. Therefore, the MD method is most widely used when numerous molecules are involved in designing MIPs, such as in optimizing the ratio of template, monomer, and cross-linking agent. The application of MM, MD, and QM methods in MIP simulation is given in Table 1.

### 2.1. MM Method

The MM method treats molecules as a collection of atoms held together by elasticity or resonance force. It uses energy functions, such as internal energy terms, including bond length, bond angle, and dihedral angle changes, to calculate changes in the molecular internal energy caused by changes in molecular structure. Combined with nonbonding energy (electrostatic interaction), these potential energy functions are called potential functions, and their parameters can be obtained by fitting quantum chemistry calculation results or experimental data. The MM method can optimize the molecular static structure of thousands of atomic systems; perform molecular structure optimization, system dynamics, and thermodynamic calculations; and select the smallest energy and the most stable molecular conformation in the space structure. However, this method ignores the movement of electrons and cannot simulate the state of electron movement in chemical reactions, thereby lowering the accuracy of the calculation [84,85,86].

Common force fields used in MIP calculation simulation by MM method are OPLS3, CHARMM, MMFF94, AMBER MM and MMFF94X. Compared with other commonly used small molecule force fields, the OPLS3 supplies reference data and related parameter types which exceed one order of magnitude. Therefore, this force field achieves a high level of accuracy in the performance benchmark for assessing conformational propensity and solvation of small molecules. It is mainly suitable for liquid systems, such as peptides, proteins, nucleic acids, and organic solvents. In addition, it employs lots of reference data and related parameter types; this also limits its use in the simulation of small molecule systems. The CHARMM force field is mainly used for the simulation of biological macromolecules, including energy minimization, molecular dynamics and Monte Carlo simulation. The simulation process provides information about molecular structure, interaction, energy, etc. The MMFF94 force field provides good accuracy in a series of organic and pharmaceutical molecular simulation calculations. The core parameterization is provided by high-quality quantum computing without a large amount of experimental data for testing molecular systems. It performs well in optimizing geometry, bond length, angle, as well electrostatic and hydrogen bonding effects. The AMBER force field also has significant and extensive applications in the field of simulation and calculation of biological macromolecules. Its advantage lies in the calculation of biological macromolecules, but the calculation results of small molecule systems are often unsatisfactory.

### 2.2. MD Method

**Figure 3:**
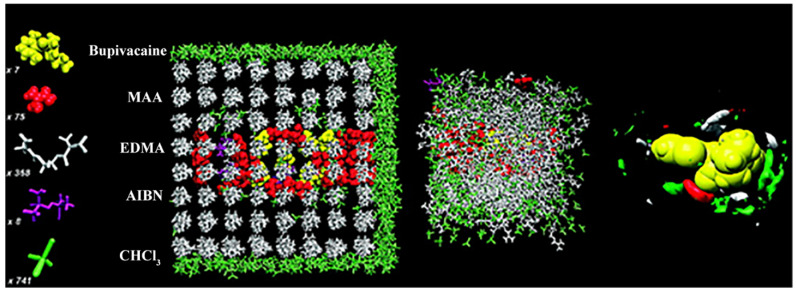
Schematic diagram of molecular dynamics calculation simulation molecular imprinting pre-assembly (Reprinted with permission from [85]. Copyright 2009 American Chemical Society).

MD methods can clarify the macroscopic properties of particles in dynamic motion. In 1957, Alder and Wainwright developed the MD technology. They computationally simulated the behavior of hard balls in boxes at different temperatures and densities. This method aims to establish a particle system via Newtonian mechanics and statistical mechanics, calculate the speed and position of molecules, obtain the state of motion of molecules, and integrate the dynamics and thermodynamic properties of systems. It is based on molecular mechanics and considers the influence of external environment, such as temperature and pressure, to calculate the molecular structures (crystallization, expansion and compression, vitrification, and deformation) and thermodynamic parameters of molecules in motion. The calculation result is close to the real state. The application of MD in molecular imprinting is illustrated in Figure 3. An MIP pre-polymerization system can possibly be established, the components and concentrations used in synthesizing the corresponding polymer copied, and the interactions and conformational changes between molecules observed.

The Tripos force field in the MD method has been proven to produce molecular geometry close to the crystal structure for different molecular selections. This force field has good calculation results in both protein and organic molecular simulations. The COMPASS force field is the first molecular force field based on ab initio calculations, which can accurately predict the molecular structure, conformation, vibration, and thermodynamic properties of isolated and condensed molecules. The COMPASS force field is also the first molecular force field that unifies the organic molecular system and the inorganic molecular system that were previously treated separately. It can simulate organic and inorganic small molecules, macromolecules, some metal ions, metal oxides and metals. The GROMOS force field guarantees the accuracy of the parameters through a series of quantum chemical calculations and existing databases. Most importantly, this force field fully considers the symmetry of the molecular structure to make the simulation more perfect.

### 2.3. QM Method

Many reports on the application of computational simulation in molecular imprinting technology have been published. Most of them used QM-based calculation methods. The primary QM simulation calculation methods are ab initio calculation methods, semiempirical calculation methods, and density functional theory (DFT) methods.

The ab initio calculation method is based on the Hartree–Fock method. This method uses some of the most basic physical constants, such as the speed of light and Planck’s constant, as known parameters, and it adopts mathematical methods to calculate molecular physics and chemistry without introducing empirical parameters. In this method, the SDCI method is applied to both the single excited and double excited states of molecules. Other methods, such as CISD(T) and MCSCF, consider different variables. The ab initio calculation method is not limited to the structure of small molecules as it can also calculate the static and dynamic properties of macromolecular systems, including intramolecular and intermolecular interactions [87,88]. The ab initio calculation method is a quantum chemical calculation method that directly solves the Schrodinger equation based on the basic principles of QM method. Compared with the semiempirical method, the ab initio calculation method is more accurate, but time-consuming.

The semiempirical method introduces some experimentally measured parameters on the basis of the ab initio calculation method, simplifies the Hartree–Fock method, and reduces the amount of experimental calculation. This method can calculate and simulate the electronic structure and properties of real biological systems, such as enzymes and proteins. Other calculation methods are available: AM1, PM3, EHMO, CNDO, and NDDO. Among these methods, AM1 can predict the existence of hydrogen bonds between molecules by calculating the activation energy of particles. However, when it is used to calculate thermodynamic properties, such as the enthalpy of particles, large errors are committed. PM3 usually has a small error, and it is used in molecular simulations and theoretical calculations [89]. However, if the target analyte is a large molecule, only semiempirical methods have practical calculation meaning. This method is often used as the first step of high-precision calculations to obtain the initial structure of subsequent calculations. Some errors are easily reported due to the use of large reference parameters from the beginning. Thus, the optimization of the basis set results in a very slow optimization speed, or there can even be failure to optimize. In general, the semiempirical method is only suitable for simple organic molecules, and qualitative information, such as molecular orbital, electric charge and normal mode, could be obtained. 

The DFT method is based on the Hohenberg–Kohn principle. It uses the electron density function in molecular simulation. The computational complexity of this method is small, and it can calculate molecular bond energy [90], predict compound structures [91], and predict reaction mechanisms [92]. Various molecular modeling and theoretical calculations in molecular imprinting technology apply the DFT method. It mainly calculates the binding energy (ΔE) between the template molecule and the functional monomer. In general, the lower the energy value is, the stronger the intermolecular interaction will be, indicating that the composite system of the template molecule and the functional monomer is more stable, which means that the prepared MIP has superior performance [93,94,95,96]. This method expresses the kinetic energy of an atom as a functional of electron density, which adds the classical expressions of the nucleus–electron and electron–electron interaction to calculate the energy of the atom. However, it is still difficult to describe the intermolecular forces, especially van der Waals forces, or the energy gap calculated by DFT method.

The calculation methods of theoretical molecular simulation used in molecular imprinting technology largely adopt the QM method. However, the QM method also has shortcomings. This method can only qualitatively predict the type of interaction between molecules but cannot accurately describe the energy changes of complex mixtures. Therefore, the previous research using this method for theoretical simulation has mostly applied to the qualitative description of molecular systems [97,98]. The amount of calculation involved in a quantitative system exponentially increases with the increase in the number of molecules, a condition that seriously affects calculation efficiency and accuracy.

## 3. Computational Simulation and Design of New MIPs

The application of theoretical calculations in designing MIPs is primarily achieved by theoretical simulations and selection of appropriate functional monomers, template molecules, crosslinkers, and their ratios. The binding energy (i.e., electronic interaction energy) between the template molecule and the functional monomer can be simulated and calculated provided that the binding energy between the template molecule and the functional monomer is high, indicating that the corresponding MIPs have excellent selectivity and adsorption performance. In addition, the ratio of the molecular and monomer system is closely related to the imprint factor of MIPs. In general, this ratio is calculated and optimized by performing the computational simulation in a vacuum environment to obtain the Eqaution (1) for the binding energy between the template molecule and the functional monomer.

*ΔE* = *E*_*(Template-monomer complex)*_ − *E*_*Template*_ − *E*_*monomer*_ (1)

In most cases, vacuum simulation calculations often differ from the actual situation as they consider the effects of spatial media, including the addition of solvents, to make the simulation calculation highly consistent with experimental results. The solvent (i.e., porogen) affects the energy of the system during the synthesis of MIPs. The results of molecular modeling can be made closer to real situation and the reliability of the results can be increased by conducting the simulation of a molecular fingerprint polymer in a solvent medium. The binding energy is calculated by Equation (2):

*ΔE*_*Solvent*_ = *E*_*(Template-monomer complex in solvent (pore-forming agent))*_ − *E*_*(template-functional complex in the gas phase)*_ (2)

where *ΔE*_*Solvent*_ is the energy difference between a template molecule and a functional monomer in solution and in a vacuum environment. A weak influence of the solution on noncovalent interactions during molecular fingerprint polymerization results in a small energy difference value, suggesting that the solvent is the best polymerization solvent for obtaining molecular fingerprint polymers [99,100].

The primary factor in MIP imprinting polymerization is the strong bonding force between the template and the functional monomer. Therefore, choosing the right functional monomer is a key factor in designing MIPs. An MIP can be reasonably designed by applying the DFT method in selecting the monomer with the best interaction with 2-isopropoxyphenol; it can be combined with the PM3.5 method to optimize the template-to-monomer ratio [101]. Quantum calculations were performed using the Spartan software, and the complexes’ binding energy can be obtained to evaluate their stability. Pyrrole had been selected as the best functional monomer for designing 2-isopropoxyphenol MIPs. PM3 and DFT calculation methods were also used to simulate and calculate the monomers with the strongest interaction with disulfoton [102], chlorogenic acid [103], and amoxicillin [104], as well as the best ratio between the two. This method can be further used to calculate the solution energies of baicalein and acrylamide complexes in different solvents to screen the best polymerization solvent [105].

**Figure 4:**
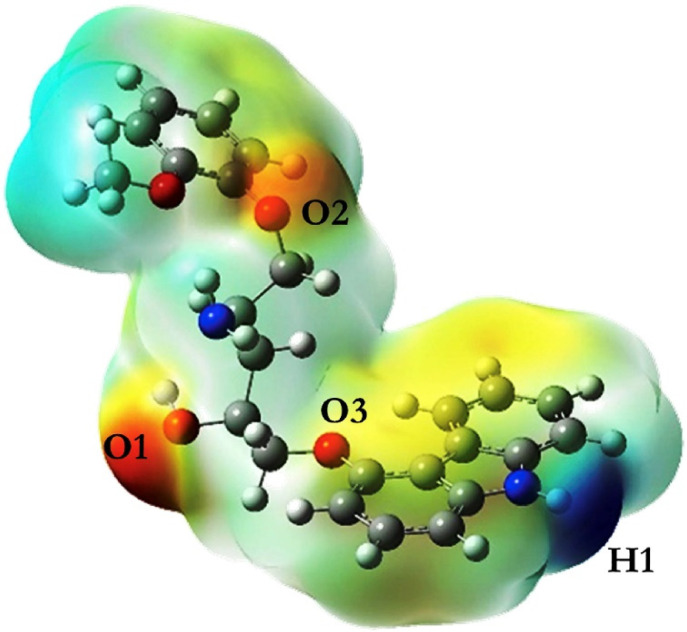
Electrostatic potential energy diagram of endotoxin in template (Reprinted with permission from [106]. Copyright 2019 Elsevier).

The strongest interaction site can be further located by obtaining the electrostatic potential map on the surface of the template molecule via the DFT method [106]. Figure 4 shows the electrostatic charge distribution of carvedilol after the geometry was optimized. The hydrogen bonding sites between carvedilol and functional monomer evidently appear in the red, yellow, and blue regions, which were O1, O2, O3, and H1. According to the quantitative information of the electrostatic map, each functional monomer undergoes hydrogen bonding at the four interaction sites in sequence to form hydrogen bonds; thus, the ratio of template and monomer complexes were 1:1 and 1:2, and 1:3 and 1:4. When the functional monomer is methacrylic acid and the template is combined with the monomer at a ratio of 1:4, a stable complex can be formed. The DFT method had also been adopted to study the interaction between p-nitrophenol and β-cyclodextrin [12].

The key to the selectivity and enrichment ability of MIPs lies in the formation of a stable complex between the template and the functional monomer. Therefore, choosing the right functional monomer is an important factor in designing MIPs. The DFT method had been employed to study intermolecular interactions between harmane and functional monomer [107]. Firstly, MD simulation was performed at constant energy by combining it with the PM3 method. Subsequently, quenching kinetics and simulated annealing were combined to perform geometric optimization calculations on the structure of the template and monomer complex. The calculated optimized energy was then compared to finding the lowest energy conformation. The DFT method calculates the frequency of the harmane–monomer (1: *n*) complex system with the smallest energy value. It obtained the theoretical parameters of intermolecular interactions and provided a reliable theoretical basis for the interaction between the template and the monomer. Similar molecular simulations of combining DFT and MD methods had been used in furazolidone [108] and kojic acid [109] imprinted polymer designs. Another work had applied the COMPASS force field in MD simulation to calculate the intermolecular interactions of ibuprofen, naproxen, and diclofenac with 2-vinylpyridine [110]. Before MD simulation, the energy of the geometric optimization of the composite system was minimized in the Materials Studio software, and the intermolecular binding energies of each system were simultaneously calculated. The influence of the solvent on the composite system was also considered, which can be seen from the optimized conformation of the calculation. During synthesis, toluene did not participate in the monomer–template interaction, indicating that the effect of toluene on the selective adsorption of such MIPs was negligible.

The DFT calculation method had also been used to explore the influence of different porogens on the binding energy of nicotinamide to monomer methacrylic acid [111]. Template molecules, functional monomers, and template-monomer complexes are modeled in a vacuum and then optimized for conformation to calculate the single-point energy of the PM3 level. When toluene is used as a porogen, a small dielectric constant and an aprotic solvent may result in a large interaction force between the template and the monomer. The MIP prepared under this condition exhibits the ideal affinity and selectivity for the target molecule. Farhad et al. [112] calculated and synthesized a new MIP of ephedrine. They used the restricted Hartree–Fock method in DFT and then applied the Podient continuum model to simulate and calculate the optimal polymerization solvent. They found that methacrylic acid and methanol were the best monomers and porogens for pseudoephedrine MIP. The calculated simulation results were consistent with the experimental results. The effect of imprinting was further maximized using the DFT method in designing and preparing clenbuterol MIP [53]. The simulation results showed that the best functional monomer was acrylic acid, and the best ratio of the template molecule clenbuterol and its norepinephrine to monomer was 1:3. Given that MeOH has the smallest solvent energy, it was selected as the best porogen. In the simultaneous calculation and simulation of various types of crosslinking agents, when ethylene glycol dimethacrylate was used as the crosslinking agent, the crosslinking agent exerts the least interference to the imprinting process. The same method has been used to study the thermodynamic properties of the polymerization process [113]. Preliminary conformational optimization of the intermolecular electrostatic potential showed that atrazine had five interaction sites. A strong complex interaction was achieved when atrazine and 2-(trifluoromethyl) acrylic acid were combined at a ratio of 1:4. As a porogen, toluene had the least interference to the self-assembly system. The calculation temperature of thermodynamic properties was within the range of 278.15–308.15 K, and the enthalpy (ΔH), free energy (ΔG), and entropy (ΔS) of the best imprinting combination system in vacuum and the toluene medium were calculated. Thermodynamic analysis revealed that atrazine MIP was beneficial to the formation of imprinting sites in a medium with a low polarity and temperature.

Baggiani [114] applied the semiempirical quantum method (AM1) to screen the best combination of six functional monomers and carbamate interactions. They used a simulated annealing algorithm to optimize the structural arrangement of supramolecules, and they calculated the heat of the formation of six composite systems. Because both acrylamide and methacrylic acid can form two different hydrogen bond interactions with the template molecule, they were considered to be the most suitable functional monomers for preparing urethane imprinted polymers. In recent years, the simulated annealing algorithm has been independently used to attain a stable conformation of the template and the monomer complex [43,44,46]. During the annealing process, the temperature of the local environment is always in a dynamic process, ensuring that the templates and the monomers located at different positions and directions are bonded, thereby expanding the available conformations. To screen suitable functional monomers from monomer library quickly, Elena [115] employed the Tripos force field and combined it with the Leapfrog algorithm to simulate and calculate the possible interactions between simazine and 20 functional monomers. Simazine and methacrylic acid achieved a high binding energy mainly through electrostatic interactions. The combination of the Tripos force field and the Leapfrog algorithm is widely used in screening functional monomers [39,40,41,116]. Owing to the ability of this combination to simulate imprinting sites visually, this method can quickly determine the monomer library. The monomer with the strongest interaction with the target molecule substantially shortens the time required to screen the best monomer from numerous monomers [117,118].

An imprinted polymer membrane that can selectively adsorb atrazine in an aqueous medium can be developed [119] using the Hyperchem software to find a functional monomer suitable for atrazine. When methacrylic acid was used as a functional monomer, two ionic bonds and three hydrogen bonds can form between atrazine and methacrylic acid. Liang et al. [120] designed and prepared a porous core–shell MIP for the selective separation and enrichment of ursodeoxycholic acid through computational simulation. They selected the molecular force field AMBER for computational simulation. The conformation of the template, monomer, and porogen are optimized, and then the MD optimization method was used to calculate the binding energy of the template and the monomer complex in the three solvents. MIP with toluene as the porogen had the strongest affinity and selectivity for ursodeoxycholic acid. Viveiros et al. [121] applied the supercritical carbon dioxide technology for the molecular imprinting of acetamide for the first time. They selected the best functional monomer, crosslinking agent, and their molar ratio for each template molecule. They then introduced CO_2_ as a solvent into the model. The theoretically designed itaconic acid MIP exhibited a high affinity and selectivity to acetamide.

Molecular simulation methods are also used in designing and synthesizing dummy template MIPs. Feng [122] adopted MM combined with AM1 to select the best alternative template for clenbuterol and its metabolites. Dummy template MIP for sulfonylurea herbicides can also be obtained by theoretical calculations by using the Hyperchem software for molecular modeling [32]. The lowest energy conformation of the template and monomer is optimized by the MM and PM3 methods, and the binding energy of the complex was obtained by AMBER MM force field. The difference between mesulfuron-methyl and nine other sulfonylurea herbicide molecules was the smallest. Thus, it was considered to be the best dummy template molecule. 

Molecular simulation calculation can be also performed in designing chiral enantioselective imprinted polymers. Sobiech [123] prepared an octopamine chiral selective MIP via computational simulations. Discovery Studio can visualize the interface to build molecular models. The DFT method can be used to optimize the geometry of all compounds, whereas its combination with the Breneman model can reproduce the atomic part electrostatic potential of each molecule. During modeling, the monomers were randomly distributed around the template, and intermolecular interactions occurred during the energy minimization process. The simulation results showed that the binding energy of octopamine and 4-vinyl benzoic acid was the smallest. Furthermore, a two-step method of MM and QM were used to simulate the design of enantioselective tBOC-tyrosine imprinted polymers [124]. The geometric structure of the molecule was greatly optimized after the two-step simulation calculations from the MM method to the MD method. This multiscale “coarse to fine” technology can address the shortcomings of using the MM method or the QM method alone and combine their advantages. The molecular structure can be roughly optimized under the MMFF94 force field. The QM method was then used to further refine its geometry. After optimizing the system, the imprinted target molecule was deleted from the QM-optimized geometry, leaving behind the “imprint” binding site. Further analysis of single monomer–target interactions in the binding sites suggested that the hydrogen atom in the chiral center would be more conducive to bonding with the functional group than the one in the chiral center. The enantioselectivity factor was obtained by determining the ratio of the binding energy of the target to the binding energy of its enantiomers. If the value is greater than 1, then the imprinted polymer can be considered to have enantioselective recognition ability for the chiral target molecule.

## 4. Computational Simulation and MIP Identification Mechanism

Theoretical simulation can also provide a theoretical basis for the identification mechanism of MIPs. The formation process of experimentally proposed magnetic molecularly imprinted polypyrrole at the molecular level can be understood via the DFT method to obtain the thermodynamic properties of the prepolymerized template and the monomer complex in the presence of water. On the basis of the negative values of ΔG and ΔH, this results in the complexation of the monomer with praziquantel in aqueous solution spontaneously forming stable complexes. Moreover, the results of molecular geometric conformation simulation showed that four hydrogen bonds and one π–π stacking interaction are established between praziquantel and pyrrole, which explains the formation of praziquantel and pyrrole prepolymer complex at the molecular level [125]. Through PM3 and DFT theoretical simulation methods, the Muliken charge on each atom of the fluazuron optimized geometric structure can be obtained, which can quantitatively reveal the existence of six regions with a high electron charge density. These local regions can interact with methacrylic acid molecules and build hydrogen bonds. If the value of enthalpy and Gibbs free energy is less than zero, then the prepolymer complex of flusulfuron–methyl and methacrylic acid can be considered to have spontaneously and stably formed. These simulation results explain the polymerization mechanism of fluazuron MIP [126].

The selective mechanism of ciprofloxacin-imprinted membrane was also further explained through molecular simulations [127]. The binding energy of the interaction between the functional monomer and ciprofloxacin and its structural analogs, including norfloxacin hydrochloride, enrofloxacin hydrochloride and ofloxacin hydrochloride, was obtained through molecular simulation calculations. Kinetic simulations had also been performed using GROMACS software. The parameters of bond, angle, dihedral angle, and Lennard–Jones interaction had been directly taken from the GAFF force field. Part of the charge was obtained using the restricted electrostatic potential method at the theoretical level of B3LYP/6-31+G (d, p). The recombination ability of the imprinted site of ciprofloxacin was dominated by hydrogen bond interactions, whereas its structural analogs were dominated by van der Waals interaction. Thus, strong hydrogen bond interactions led to a high tendency for the imprinted site of ciprofloxacin to recombine with the template molecule. Theoretical simulations of the recombination mechanism and selective permeation experiments mutually confirmed the superior selectivity of ciprofloxacin-imprinted membranes. Zhang [128] further explained the specific selective recognition mechanism of molecularly imprinted nanocomposite membranes for artemisinin by dynamic simulations. A comparison of the binding energy of the imprinted membrane with artemisinin and its structural analogs shows that the strong interaction between artemisinin and the imprinted polymer matrix contributes to its large adsorption capacity and high selectivity. A similar DFT method has been used to explain the selective recognition mechanism of the alternative template *N*-(4-isopropylphenyl)-*N′*-butyleneurea MIP to phenylurea herbicides [129]. This method also explained the mechanism of experimentally preferred dummy template imprinted polymer [130] and the strength of the bonding force of chiral naproxen MIP [21] at the molecular level. These observations provide a theoretical basis to explain the experimental results from the perspective of intermolecular interactions.

Yang [61] performed molecular simulation to reveal the essential reason for the difference between single-template and double-template MIP stirring bars in their ability to recognize target analytes by using the YASARA software to study the recognition mechanism. The 3D shape and size of the imprinted cavity in the MIPs are the corresponding template molecules. Given that the dual-template MIP contains imprinted cavities of the two template molecules, it had a fairly high recovery for nine fluoroquinolones, and the simulation results are consistent with the experimental findings. However, the influence of template–template interactions on the performance of multitemplate MIPs has been further verified via the DFT method [62]. The results of both theoretical simulations and experiments indicated that the interaction between more template molecules affects the formation of specific recognition sites and even reduces the formation of effective imprinting sites.

## 5. Conclusions and Outlook

Computer molecular modeling technology has been applied to the screening and optimization of molecules in many materials, and it is also a feasible method for preliminary exploration of MIP. Computer simulation reduces the time and reagent-related costs required to obtain the appropriate MIP adsorbent, and significantly reduces the consumption of organic solvents. In addition, it can explain the specific recognition mechanism of imprinted materials at the molecular level. For all the above reasons, the use of computer molecular simulation to design MIP adsorbents in analytical practice not only conforms to the principles of green analytical chemistry, but also explains the nature of MIPs binding to target molecules from the intermolecular forces. The QM method, compared with other methods, can ensure more accurate simulation results in the MIP system dominated by non-bonding interaction, because the smallest structural unit electron was studied and the quantum effect was considered in the method. Therefore, the QM method is also the most widely used in MIP simulation operations. However, in the simulation of macromolecules and polyatomic systems, this method is very time-consuming and even prone to errors. MM and MD are classical mechanics methods. Their smallest structural unit is no longer an electron but an atom. Therefore, the simulation operation complexity of the imprinting system is greatly reduced, and the operation speed is faster. MM method directly utilizes the potential function to study the problem, without considering the kinetic energy and the corresponding structure of the atom. However, the MD method focuses on the movement of atoms in the MIP system and establishes the relationship between temperature and time, which can simulate the imprinting system more realistically, and the simulation results are more representative. In general, the DFT procedure in the QM method was recommended in the MIPs design and mechanism interpretation simulation calculation. However, this also means that the computational complexity of this method increases dramatically for large molecules and systems with a large number of molecules. MD method may be the best solution at this situation, simulated annealing process in particular, which can complete the lowest energy conformation search in a very short time. At present, an increasing number of research have been using multiple calculation methods to achieve complementary advantages when designing and optimizing the experimental parameters of MIPs preparation, so as to ensure more efficient and accurate simulation results. In addition, simulation is also the direction of current efforts. A more realistic simulation environment can make the calculation results accurate and reliable.

## Figures and Tables

**Table 1:** Theoretical simulation calculation methods for the design of MIPs.

Simulation Method	Template	Force Field/Method	Software	MIPs Design
*Molecular mechanics (MM)*	Myoglobin [29]	OPLS3	Prime	Screening functional monomers
	Morphine [30]	CHARMM and MMFF94	Discovery Studio	Template-monomer ratio
	Metolachlor deschloro [31], metsulfuron-methyl [32]	AMBER MM	SPSS Statistics	Screening functional monomers/template-monomer ratio
	Norfloxacin [33]	MMFF94X	Discovery Studio	Screening functional monomers/template-monomer ratio
*Molecular dynamics (MD)*	Curcumin [34], fenthion [35], *N*-3-oxo-dodecanoyl-L-homoserine lactone [36], methidathion [37], endotoxins [38], phosmet insecticide [39], cocaine [40], methyl parathion [41], aflatoxin B1 [42]	Tripos	SYBYL	Screening functional monomers/template-monomer ratio
	Bisphenol A [43], carbamazepine [44], phthalates [45], norfloxacin [46], sulfamethoxazole [47]	COMPASS	Materials Studio/accelrys.com	Screening functional monomers/template-monomer ratio
	Thiamethoxam [48]	AMBER	Gaussian	Template-monomer ratio and solvent
	Rhodamine B [49]	GROMOS	GROMACS	Template-monomer ratio and solvent
*Quantum mechanics (QM)*	Vancomycin [50], primaquine [51], tramadol [52], thiamethoxam [48], clenbuterol [53], sulfadimidine [54], bilobalide [55], chloramphenicol [56], paclitaxel [57], acetamiprid [58], acetazolamide [59], lamotrigine [60], cyanazine [61], 3-methylindole [62], polybrominated diphenyl ethers [63], pirimicarb [64], metoprolol [65], ciprofloxacin or norfloxacin [66]	DFT	Gaussian	Screening functional monomers/template-monomer ratio/solvent
	Aspartame [67], pinacolyl methylphosphonate [68], metolachlor deschloro [31], metsulfuron-methyl [32], thiocarbohydrazide [69]	Semiempirical method	Spartan/SPSS Statistics	Screening functional monomers/template-monomer ratio
	Benzo[a]pyrene [70], tryptophan [71], furosemide [72], buprenorphine [73], hydroxyzine and cetirizine [74], atenolol [75], diazepam [76], metolachlor deschloro [31], metsulfuron-methyl [32], allopurinol [77], methadone [78], clonazepam [79], theophylline [80], ametryn [81], mosapride citrate [82], baicalein [83],	Ab initio	HyperChem/Gaussian/AutoDockTools/SPSS Statistics	Screening functional monomers/template-monomer ratio

## Data Availability

There are no data associated with this publication.

## Abbreviations

MIP	Molecularly imprinted polymers
MM	Molecular mechanics
MD	Molecular dynamics
QM	Quantum mechanics
DFT	Density functional theory
OPLS	Optimized potentials for liquid simulations
CHARMM	Chemistry at Harvard macromolecular mechanics
MMFF	Merck molecular force field
Tripos	Tripos force field
COMPASS	Computer plan appraisal system for ship
GROMOS	Gromos force field
Ab initio	Latin term meaning “from the beginning”
B3LYP	Becke, three-parameter
GAFF	Generation amber force field
SDCI	Single and double excitation configuration interaction
CISD(T)	Configuration interactions with single and double substitutions
MCSCF	Multiconfiguration self-consistent field
AM1	AM1 semiempirical method
PM3	Semiempirical PM3 method
EHMO	Extended huckel molecular orbital theory
CNDO	Complete neglect of differential overlap method
NDDO	Neglect of diatomic differential overlap method
